# Correlation between Selenium and Zinc Levels and Survival among Prostate Cancer Patients

**DOI:** 10.3390/nu16040527

**Published:** 2024-02-14

**Authors:** Sandra Pietrzak, Wojciech Marciniak, Róża Derkacz, Milena Matuszczak, Adam Kiljańczyk, Piotr Baszuk, Marta Bryśkiewicz, Andrzej Sikorski, Jacek Gronwald, Marcin Słojewski, Cezary Cybulski, Adam Gołąb, Tomasz Huzarski, Tadeusz Dębniak, Marcin R. Lener, Anna Jakubowska, Tomasz Kluz, Rodney J. Scott, Jan Lubiński

**Affiliations:** 1 Department of Genetics and Pathology, International Hereditary Cancer Center, Pomeranian Medical University in Szczecin, ul. Unii Lubelskiej 1, 71-252 Szczecin, Poland; sandra.pietrzak@pum.edu.pl (S.P.); milena.matuszczak@pum.edu.pl (M.M.); adam.kiljanczyk@pum.edu.pl (A.K.); piotr.baszuk@pum.edu.pl (P.B.); marta.bryskiewicz@pum.edu.pl (M.B.); jacek.gronwald@pum.edu.pl (J.G.); cezarycy@pum.edu.pl (C.C.); tomasz.huzarski@pum.edu.pl (T.H.); tadeusz.debniak@pum.edu.pl (T.D.); marcin.lener@pum.edu.pl (M.R.L.); anna.jakubowska@pum.edu.pl (A.J.); 2 Read-Gene, Grzepnica, ul. Alabastrowa 8, 72-003 Dobra, Poland; wojciech.marciniak@read-gene.com (W.M.); roza.derkacz@read-gene.com (R.D.); 3 Department of Urology and Urological Oncology, Pomeranian Medical University in Szczecin, al. Powstańców Wielkopolskich 72, 71-899 Szczecin, Poland; sikor@post.pl (A.S.); marcin.slojewski@pum.edu.pl (M.S.); adam.golab@pum.edu.pl (A.G.); 4 Department of Clinical Genetics and Pathology, University of Zielona Góra, ul. Zyty 28, 65-046 Zielona Góra, Poland; 5 Department of Gynecology, Gynecology Oncology and Obstetrics, Fryderyk Chopin University Hospital No. 1, 35-055 Rzeszow, Poland; jtkluz@interia.pl; 6 Institute of Medical Sciences, Medical College of Rzeszow University, 35-959 Rzeszow, Poland; 7 Priority Research Centre for Cancer Research, Innovation and Translation, Hunter Medical Research Institute, New Lambton, NSW 2305, Australia; rodney.scott@newcastle.edu.au; 8 School of Biomedical Sciences and Pharmacy, Faculty of Health and Medicine, University of Newcastle, Callaghan, NSW 2308, Australia; 9 Division of Molecular Medicine, Pathology North, John Hunter Hospital, New Lambton, NSW 2305, Australia

**Keywords:** selenium, zinc, prostate cancer, survival

## Abstract

The most prevalent type of cancer among males is prostate cancer. Survival is considered quite good, but it can be further improved when risk factors are optimized. One of these factors is micronutrients, including Se and Zn. To our knowledge, the interaction between Se and Zn and prostate cancer remains undescribed. This study aimed to investigate the optimal levels of selenium (Se) and zinc (Zn) and their impact on the survival of individuals diagnosed with prostate cancer. A total of 338 prostate cancer patients were enrolled in this study, which was conducted in Poland between 2009 and 2015. Mass spectrometry, which uses inductively coupled plasma mass, was used to assess serum element levels before treatment. The study participants were categorized into quartiles (QI-QIV) based on the distributions of Se and Zn levels observed among surviving participants. Cox regression was used to assess the association between serum Se and Zn levels and the survival of prostate cancer patients. Our results reveal the effect of combined Se and Zn levels on survival in prostate cancer patients (SeQI-ZnQI vs. SeQIV-ZnQIV; HR = 20.9). These results need further research to establish Se/Zn norms for different populations.

## 1. Introduction

Prostate cancer ranks as the most prevalent cancer in men and stands as the second primary cause of mortality. In the year 2020, a worldwide assessment reported 1,414,259 newly diagnosed cases and 375,304 deaths linked to this particular malignancy [1]. Survival is relatively high, as demonstrated by the EUROCARE-5 study, which revealed an overall 5-year survival rate of 83% [2]; for US patients, the 5-year survival is even better at ~97% [3], but it could still be improved.

A notable problem in the management of prostate cancer involves the identification of the determinants affecting survival. Generally, factors affecting survival vary between regions and cultures and can interact and complement each other. The most frequent of these include cardiovascular diseases, diabetes, obesity, respiratory insufficiency/illnesses [4], disease progression, lifestyle choices (alcohol/tobacco use, physical activity levels), and environmental factors. Among the plethora of environmental factors influencing survival are some elements that have been studied, such as iron (Fe) [5,6,7], cadmium (Cd) [8], mercury (Hg) [8], selenium (Se) [9,10,11,12,13,14,15,16], and zinc (Zn) [17].

Se is a crucial element necessary for the optimal physiological functioning of diverse organisms. The exact mechanisms by which serum Se influences survival remain uncertain, and the association between an unfavorable prognosis and diminished Se levels is a subject of contention. To date, Se has been shown to be associated with survival in several malignancies, which include laryngeal, breast, lung, and colorectal cancers and malignant melanoma [9,10,11,12,13,14,15,16,17,18,19].

Zn is an essential trace element widely distributed throughout the environment that plays a pivotal role in human metabolism. Moreover, Zn is a required cofactor for the activation of over 300 enzymes. Additionally, it forms an integral part of structural and regulatory proteins, including transcription factors, establishing “zinc fingers” that facilitate DNA binding. Zn levels have been linked to survival in prostate, breast, lung, and laryngeal cancers [17,20,21].

It is well-established that the effect of a single element can vary depending on the levels of other micronutrients. Molecular and cellular investigations suggest that Se and Zn exhibit reciprocal effects influenced by variations in Zn level [22]. The low-dose Se and Zn supplementation model can be used to reduce the risk of prostate cancer and overall mortality [21,23,24]. However, it should be remembered that supplementation should be performed carefully after studying the level of micronutrients in the patient’s blood/serum. The optimal level should be targeted, because both deficiency and excess can adversely affect the patient’s prognosis [25,26,27,28].

To the best of our knowledge, the interaction between Se and Zn and cancer survival remains undescribed. Hence, our study aimed to examine the optimal levels of Se and Zn and their contribution to survival in individuals diagnosed with prostate cancer.

## 2. Methods and Materials

### 2.1. Study Cohort

A total of 338 unselected patients with prostate cancer were enrolled in this prospective study. The diagnosis of prostate cancer was always based on the result of histopathological examination. Cases originated from the Department of Urology and the Urological Clinical Hospital of the Pomeranian Medical University in Szczecin. Blood samples were taken from cases shortly after diagnosis and prior to treatment (between 2009 and 2015). All blood samples were collected fasting. All patients with complete information regarding their age at diagnosis (≤60/>60), Gleason (<7/7/>7), PSA (<4/4–10/>10), and vital status during follow up (alive/dead) were taken into account in the final calculations. Ethical approval for this study was obtained from the Ethics Committee of the Pomeranian Medical University in Szczecin under the reference number KB-0012/73/10 dated 21 June 2010, and the research adhered to the principles outlined in the Helsinki Declaration. Written and informed consent was obtained from all enrolled participants.

### 2.2. Methodology for Measurements

#### 2.2.1. Sample Storage and Collection

Serum samples were collected using the Vacutainer^®^ System (BD, Plymouth, UK). Blood for the serum was collected in tubes with a clot activator, incubated for a minimum of 30 min at room temperature for clotting, and then centrifuged at 1300× *g* for 12 min. The obtained serum was aliquoted into new cryovials and stored at −80 °C until analysis. On the analysis day, sera were thawed, vortex-mixed, and centrifuged at 5000× *g* for 5 min.

#### 2.2.2. Measurement Methodology

Determination of ^80^Se and ^66^Zn was conducted using an inductively coupled plasma (ICP) mass spectrometer ELAN DRC-e (PerkinElmer, Concord, Ontario, Canada). Calibration of the instrument was performed daily, and oxygen served as the reaction gas. The spectrometer was calibrated using an external calibration technique with freshly prepared daily standards from Multi-Element Calibration Standard 3 (PerkinElmer Pure Plus, Shelton, CT, USA). A 30-fold dilution of serum in a blank reagent was assumed for the analysis, consisting of high-purity water, TMAH (AlfaAesar, Kandel, Germany), Triton X-100 (PerkinElmer, Shelton, CT, USA), n-butanol (Merck, Darmstadt, Germany), and EDTA (Merck, Darmstadt, Germany). Matrix-matched calibration was performed.

#### 2.2.3. Quality Control

The precision and accuracy of measurements were assessed using certified reference material (CRM), Clincheck Plasmonorm Serum Trace Elements Level 1 (Recipe, Munich, Germany).

### 2.3. Statistical Analysis

The study cohort (*n* = 338) was categorized into quartiles (QI-QIV) based on Se and Zn serum levels within the subgroup of living patients (*n* = 246). Fourth quartiles (QIV) were chosen as the reference group. The characteristics of the study group were analyzed using the chi-square and Kruskal–Wallis tests for qualitative and quantitative data, respectively. Data normality was assessed using the Anderson–Darling test. Univariable and multivariable COX proportional hazard regression models were calculated to estimate the association between serum Se and Zn levels and prostate cancer survival considering certain variables, such as age at diagnosis (≤60/>60), Gleason (<7/7/>7), and PSA (<4/4–10/>10). The survival of alive patients was the difference between the final follow-up date (20 July 2022) and the date of prostate cancer diagnosis. The survival of deceased patients was the difference between the date of death and the date of diagnosis. For calculation purposes, a survival time longer than or equal to 5 years was treated as exactly 5 years of observation time. Kaplan–Meier curves were utilized to present univariable survival based on Se and Zn serum levels. All calculations were performed and all graphics were created using the R statistical environment (R Foundation for Statistical Computing, Vienna, Austria 2023; R version: 4.3.2).

## 3. Results

The characteristics of the study group are shown in Table 1.

The median Se level for the entire group (*n* = 338) was 76.85 µg/L (IQR = 15.55), with a mean Se level of 77.97 µg/L (±2.16). The median Zn level for the entire group was 830.40 µg/L (IQR = 169.26), and the mean level of Zn was 845.12 µg/L (±135.90).

**Figure 1:**
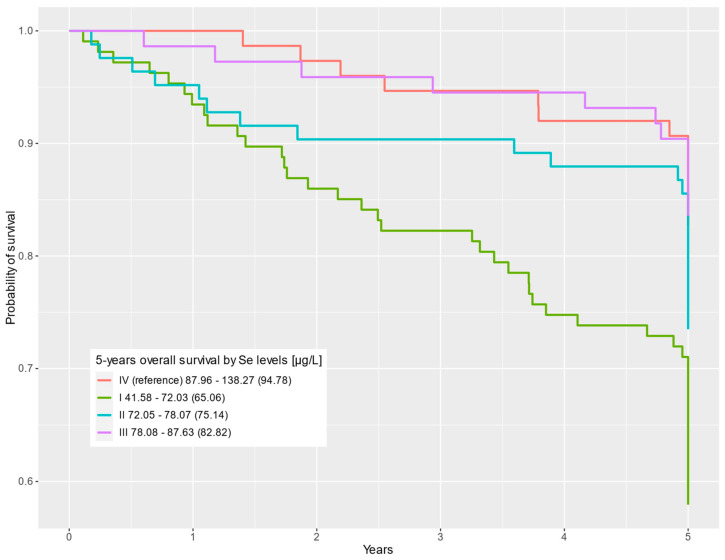
Five-year overall survival by Se level (µg/L) categorized into quartiles (QI–QIV).

**Figure 2:**
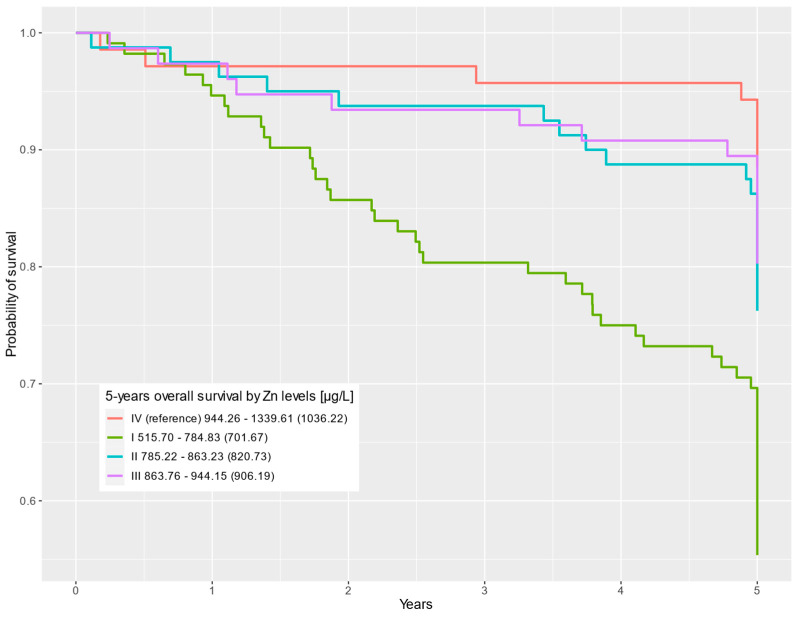
Five-year overall survival by Zn level (µg/L) categorized into quartiles (QI–QIV).

In the multivariable Cox regression, statistically significant differences were observed for the first-quartile Se level compared to the fourth Se quartile (HR = 2.43; 95% CI = 1.29–4.57; *p* = 0.006). Statistically significant differences in survival were also observed for the first Zn quartile compared to the highest Zn levels in the fourth quartile (HR = 4.11; 95% CI = 1.93–8.74; *p* < 0.001). The results for the uni- and multivariable Cox regression analyses are shown in Table 2. Survival curves depending on Se and Zn levels are presented in Figure 1 and Figure 2, respectively.

Among the total number of 338 prostate cancer patients, there were 77 (22.8%) patients with corresponding extreme quartiles of Se and Zn, whereas 49 (14.5%) patients had corresponding first quartiles of Se and Zn (SeQI-ZnQI) and 28 (8.28%) patients had corresponding fourth quartiles of Se and Zn (SeQIV-ZnQIV).

In the multivariable model, our findings show that individuals who were in both the lowest Se quartile and the lowest Zn quartile (SeQI-ZnQI) had almost a 21-fold lower chance of 5-year survival compared to patients in the highest Se and Zn quartiles (SeQIV-ZnQIV) (HR = 20.9; 95% CI = 2.80–156; *p* = 0.003).

**Figure 3:**
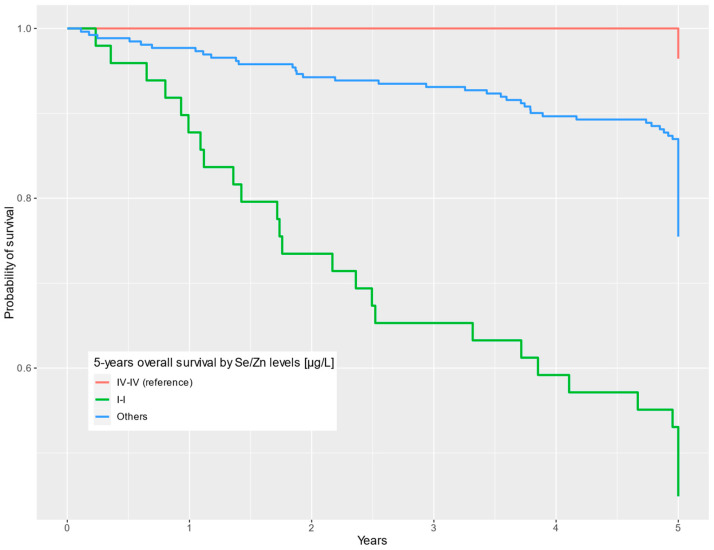
Five-year overall survival by Se/Zn levels (µg/L) categorized into common quartiles.

Results for uni- and multivariable Cox analyses are presented in Table 3. Survival curves depending on Se and Zn levels combined are presented in Figure 3.

## 4. Discussion

Some key elements have been associated with cancer risk and progression. For this reason, research is being conducted to determine if they can be used as survival markers. Our previous reports examining survival in breast, lung, and laryngeal cancer and malignant melanoma patients showed an increased risk of death with low Se levels [9,10,11,12,13]. Several previously published studies have found a moderate correlation between diminished serum Se levels and survival outcomes in individuals diagnosed with breast and colorectal cancers [15,16]. We also found that high Zn levels correlated with prolonged survival in laryngeal [17], lung, breast, and prostate cancer patients [20]. Likewise, low Fe levels can increase the risk of death, as we have shown among a cohort of patients with malignant melanoma and lung cancer [5,12]. In addition, low Fe levels slightly increase the likelihood of mortality in individuals diagnosed with oral cancer [6]. We reported that blood Cd levels below 1.97 μg/L and Hg levels below 0.44 μg/L showed a connection with increased survival rates among individuals diagnosed with stage IA lung cancer [8]. In Table 4, we have listed selected studies (inclusion criteria: study cohort *n* ≥ 100, HR > 1.3) examining the effect of micronutrient levels on cancer survival.

In the present investigation, our team evaluated whether the Se and Zn serum levels’ combined effect could be related to the survival of patients with prostate cancer.

The precise mechanisms through which these elements affect prognosis are not fully understood. Se and Zn levels can certainly serve as biomarkers, although it cannot be excluded that they contribute directly to the progression of disease. Se and Zn are involved in various metabolic mechanisms that could potentially impact prognosis. It is plausible that the progression may be driven by the activity of conjugate proteins, with metal levels potentially playing a role in facilitating this process. Se, through its incorporation into selenoproteins, contributes to maintaining cellular redox balance, which is closely connected to MAPK signaling. Similarly, recent findings have linked Zn to MAPK signaling and the oncogene BRAF, which is relevant to prostate cancer [29,30,31].

The relationship between Zn and Se and many processes involved in cancer progression has been extensively studied (Table 5). It seems that, generally, micronutrients act dependently on one another. Actually, in our studies, the correlation between Se and Zn levels is moderate (correlation coefficient = 0.32; *p* < 0.001), and for this reason, their effects appear multiplicative. However, it cannot be excluded that in the same processes involving Zn and Se, they act in opposite directions. The relationship between overall survival or cancer progression and serum Se and/or Zn levels is poorly described in the literature. To date, some reports about micronutrients affecting survival have been forthcoming, but so far, no combined effects of Se and Zn have been reported with respect to cancer progression. In this report, we would like to draw attention to the importance of the potential benefits of optimizing these essential micronutrients and implementing this information into daily life/clinical practice. Our study, as far as we are aware, is the initial report of this correlation. The results we present (SeQI-ZnQI vs. SeQIV-ZnQIV; HR = 20.9; 95% CI = 2.80–156; *p* = 0.003) point towards a tremendous potential for improving patient outcomes.

## 5. Conclusions

Our results show the impact of combined Se and Zn levels on survival in prostate cancer patients. Even though the effect of Zn has already been well-established, our data strongly indicate that it is more beneficial to optimize both Se and Zn levels. Therefore, we are going to establish a trial to prove this statement. Certainly, such a trial should be based on careful, systematic measurements of Se and Zn serum levels. At the same time, we want to draw the attention of scientists around the world to conduct similar studies to determine the best element levels for people in different regions of the world, knowing that background levels of Se and Zn vary from continent to continent.

## 6. Patents

Based on the results presented in the following paper, a patent application has been submitted to the Patent Office of the Republic of Poland (application ID P.446712).

## Figures and Tables

**Table 1:** Characteristics of the study population (*n* = 338 prostate cancer patients).

	Se						Zn				
Variable	Overall, *n* = 338	QI 41.58–72.03 (65.06), *n* = 107	QII 72.05–78.07 (75.14), *n* = 83	QIII 78.08–87.63 (82.82), *n* = 73	QIV (Reference) 87.96–138.27 (94.78), *n* = 75	*p*	QI 515.70–784.83 (701.67), *n* = 112	QII 785.22–863.23 (820.73), *n* = 80	QIII 863.76–944.15 (906.19), *n* = 76	QIV (Reference) 944.26–1339.61 (1036.22), *n* = 70	*p*
Status						<0.001					<0.001
Alive	246 (73%)	62 (58%)	61 (73%)	61 (84%)	62 (83%)		62 (55%)	61 (76%)	61 (80%)	62 (89%)	
Dead	92 (27%)	45 (42%)	22 (27%)	12 (16%)	13 (17%)		50 (45%)	19 (24%)	15 (20%)	8 (11%)	
Age						0.025					0.059
≤60 (reference) 41.00–60.00 (56.57)	77 (23%)	17 (16%)	22 (27%)	13 (18%)	25 (33%)		16 (14%)	20 (25%)	20 (26%)	21 (30%)	
>60 61.00–86.00 (68.45)	261 (77%)	90 (84%)	61 (73%)	60 (82%)	50 (67%)		96 (86%)	60 (75%)	56 (74%)	49 (70%)	
Gleason						0.3					0.5
<7	114 (34%)	43 (40%)	22 (27%)	28 (38%)	21 (28%)		43 (38%)	25 (31%)	27 (36%)	19 (27%)	
7	166 (49%)	46 (43%)	49 (59%)	33 (45%)	38 (51%)		47 (42%)	41 (51%)	37 (49%)	41 (59%)	
>7	58 (17%)	18 (17%)	12 (14%)	12 (16%)	16 (21%)		22 (20%)	14 (18%)	12 (16%)	10 (14%)	
PSA						0.2					0.2
<4	19 (5.6%)	5 (4.7%)	5 (6.0%)	5 (6.8%)	4 (5.3%)		6 (5.4%)	3 (3.8%)	7 (9.2%)	3 (4.3%)	
4–10	185 (55%)	48 (45%)	47 (57%)	47 (64%)	43 (57%)		53 (47%)	44 (55%)	47 (62%)	41 (59%)	
>10	134 (40%)	54 (50%)	31 (37%)	21 (29%)	28 (37%)		53 (47%)	33 (41%)	22 (29%)	26 (37%)	
Prostatectomy						0.001					<0.001
No	68 (20%)	31 (29%)	16 (19%)	11 (15%)	10 (13%)		34 (30%)	17 (21%)	12 (16%)	5 (7.1%)	
Yes	250 (74%)	63 (59%)	64 (77%)	60 (82%)	63 (84%)		65 (58%)	60 (75%)	62 (82%)	63 (90%)	
Missing	20 (5.9%)	13 (12%)	3 (3.6%)	2 (2.7%)	2 (2.7%)		13 (12%)	3 (3.8%)	2 (2.6%)	2 (2.9%)	
Radiotherapy						0.092					0.023
No	149 (44%)	39 (36%)	40 (48%)	32 (44%)	38 (51%)		45 (40%)	31 (39%)	40 (53%)	33 (47%)	
Yes	146 (43%)	46 (43%)	34 (41%)	35 (48%)	31 (41%)		43 (38%)	41 (51%)	30 (39%)	32 (46%)	
Missing	43 (13%)	22 (21%)	9 (11%)	6 (8.2%)	6 (8.0%)		24 (21%)	8 (10%)	6 (7.9%)	5 (7.1%)	
Chemotherapy						0.011					0.007
No	257 (76%)	68 (64%)	66 (80%)	57 (78%)	66 (88%)		75 (67%)	61 (76%)	62 (82%)	59 (84%)	
Yes	20 (5.9%)	11 (10%)	4 (4.8%)	4 (5.5%)	1 (1.3%)		4 (3.6%)	7 (8.8%)	6 (7.9%)	3 (4.3%)	
Missing	61 (18%)	28 (26%)	13 (16%)	12 (16%)	8 (11%)		33 (29%)	12 (15%)	8 (11%)	8 (11%)	
Hormonotherapy						0.035					0.10
No	197 (58%)	49 (46%)	50 (60%)	48 (66%)	50 (67%)		55 (49%)	46 (58%)	51 (67%)	45 (64%)	
Yes	107 (32%)	41 (38%)	24 (29%)	21 (29%)	21 (28%)		39 (35%)	27 (34%)	20 (26%)	21 (30%)	
Missing	34 (10%)	17 (16%)	9 (11%)	4 (5.5%)	4 (5.3%)		18 (16%)	7 (8.8%)	5 (6.6%)	4 (5.7%)	

Q—quartile; PSA—prostate specific antigen.

**Table 2:** Survival of prostate cancer patients according to serum Se and Zn levels.

	Frequency			Univariable Cox Regression			Multivariable Cox Regression		
Variable	Overall, *n* = 338	Alive, *n* = 246	Dead, *n* = 92	HR	95% CI	*p*	HR	95% CI	*p*
**Se**									
Se level									
QI 41.58–72.03 (65.06)	107 (32%)	62 (25%)	45 (49%)	2.94	1.59, 5.45	<0.001	2.43	1.29, 4.57	0.006
QII 72.05–78.07 (75.14)	83 (25%)	61 (25%)	22 (24%)	1.63	0.82–3.24	0.2	1.84	0.92–3.67	0.085
QIII 78.08–87.63 (82.82)	73 (22%)	61 (25%)	12 (13%)	0.95	0.43–2.08	0.9	1.01	0.45–2.23	>0.9
QIV (reference) 87.96–138.27 (94.78)	75 (22%)	62 (25%)	13 (14%)	—	—		—	—	
Age									
≤60 (reference) 41.00–60.00 (56.57)	77 (23%)	66 (27%)	11 (12%)	—	—		—	—	
>60 61.00–86.00 (68.45)	261 (77%)	180 (73%)	81 (88%)	2.37	1.26–4.45	0.007	2.04	1.07–3.87	0.030
Gleason									
<7	114 (34%)	80 (33%)	34 (37%)	—	—		—	—	
7	166 (49%)	135 (55%)	31 (34%)	0.60	0.37–0.98	0.040	0.62	0.38–1.01	0.056
>7	58 (17%)	31 (13%)	27 (29%)	1.84	1.11–3.04	0.018	1.54	0.92–2.59	0.10
PSA									
<4	19 (5.6%)	13 (5.3%)	6 (6.5%)	—	—		—	—	
4–10	185 (55%)	156 (63%)	29 (32%)	0.45	0.19–1.09	0.076	0.50	0.21–1.21	0.12
>10	134 (40%)	77 (31%)	57 (62%)	1.46	0.63–3.38	0.4	1.32	0.56–3.08	0.5
**Zn**									
Zn level									
QI 515.70–784.83 (701.67)	112 (33%)	62 (25%)	50 (54%)	4.91	2.33–10.4	<0.001	4.11	1.93–8.74	<0.001
QII 785.22–863.23 (820.73)	80 (24%)	61 (25%)	19 (21%)	2.22	0.97–5.08	0.058	2.08	0.91–4.75	0.084
QIII 863.76–944.15 (906.19)	76 (22%)	61 (25%)	15 (16%)	1.81	0.77–4.27	0.2	1.87	0.79–4.42	0.2
QIV (reference) 944.26–1339.61 (1036.22)	70 (21%)	62 (25%)	8 (8.7%)	—	—		—	—	
Age									
≤60 (reference) 41.00–60.00 (56.57)	77 (23%)	66 (27%)	11 (12%)	—	—		—	—	
>60 61.00–86.00 (68.45)	261 (77%)	180 (73%)	81 (88%)	2.37	1.26–4.45	0.007	1.87	0.99–3.54	0.053
Gleason									
<7	114 (34%)	80 (33%)	34 (37%)	—	—		—	—	
7	166 (49%)	135 (55%)	31 (34%)	0.60	0.37–0.98	0.040	0.66	0.41–1.08	0.10
>7	58 (17%)	31 (13%)	27 (29%)	1.84	1.11–3.04	0.018	1.52	0.91–2.53	0.11
PSA									
<4	19 (5.6%)	13 (5.3%)	6 (6.5%)	—	—		—	—	
4–10	185 (55%)	156 (63%)	29 (32%)	0.45	0.19–1.09	0.076	0.53	0.22–1.30	0.2
>10	134 (40%)	77 (31%)	57 (62%)	1.46	0.63–3.38	0.4	1.54	0.66–3.58	0.3

HR—hazard ratio; CI—confidence interval; Q—quartile; PSA—prostate specific antigen.

**Table 3:** Prostate cancer patients’ survival—combined effect of Se and Zn blood levels by quartiles.

					Univariable Cox Regression Models			Multivariable Cox Regression Models		
Quartile No.	Se Level (µg/L)	Zn Level (µg/L)	Alive	Dead	HR	95% CI	*p*	HR	95% CI	*p*
**SeQI-ZnQI vs. SeQIV-ZnQIV**										
**SeQI-ZnQI**	41.58–71.79	515.70–784.83	22	27	24.5	3.32–180	0.002	20.9	2.80–156	0.003
**Others**	52.77–138.27	541.75–1339.61	197	64	7.71	1.07–55.6	0.043	6.52	0.90–47.2	0.063
**SeQIV-ZnQIV**	88.78–104.22	946.99–1225.28	27	1	—	—		—	—	

HR—hazard ratio; CI—confidence interval; SeQ—selenium quartile; ZnQ—zinc quartile.

**Table 4:** Levels of selected micronutrients and their impact on cancer survival.

Study	Group (*n*)	Element	Survival	Cancer	Sample
Kornitzer et al. 2003 [14]	139	Se (≤72 vs. ≥85 μg/L *)	HR = 2.2; 95% CI = 1.3–3.7; *p* = 0.011	All	Blood serum
Lubiński et al. 2018 [9]	296	Se (<50 vs. >66.8 μg/L *)	HR = 3.07; 95% CI = 1.59–5.94; *p* = 0.0009	Laryngeal	Blood serum
Lubiński et al. 2018 [10]	546	Se (<81.0 vs. >81.0 μg/L *)	HR = 2.49; 95% CI = 1.53–4.04; *p* = 0.0002	Breast	Blood serum
Pietrzak et al. 2019 [11]	302	Se (<57.91 vs. >69 μg/L *)	HR = 2.73; 95% CI = 1.21–6.11; *p =* 0.01)	Lung	Blood serum
Sandsveden et al. 2020 [15]	1066	Se (≤81 vs. ≥100.01 μg/L *)	HR = 1.67; 95% CI = 0.37–0.98	Breast	Blood serum
Baker et al. 2021 [16]	995	Se (≤67.5 vs. ≥100 μg/L *)	HR = 1.37; 95% CI = 0.98–1.92; *p =* 0.06	Colorectal	Blood serum
Rogoża-Janiszewska et al. 2021 [12]	375	Se (<76.23 vs. >96.15 μg/L *)	HR = 5.83; 95% CI = 1.32–25.8; *p =* 0.02	Melanoma	Blood serum
Szwiec et al. 2021 [13]	538	Se (52.1–76.7 vs. 94.7–171.5 μg/L *)	HR = 2.31; 95% CI = 1.24–4.31; *p =* 0.008	Breast	Blood serum
Lubiński et al. 2021 [17]	300	Zn (<579 vs. >688 μg/L *)	HR = 2.32;95% CI = 1.47–3.69; *p* < 0.01	Laryngeal	Blood serum
Sukiennicki et al. 2021 [5]	200	Fe (<959.92 vs. >1628.62μg/L *)	HR = 1.67; 95% CI = 0.96–2.86; *p =* 0.07	Lung	Blood serum
Lin et al. 2021 [6]	747	Fe (≤15.3 vs. >15.3 μmol/L *)	HR = 1.39; 95% CI = 1.11–1.92	Oral	Blood serum
Rowińska et al. 2022 [7]	375	Fe (<893.05 vs. ≥1348.63 μg/L *)	HR = 4.66; 95% CI = 1.28–16.9; *p =* 0.019	Melanoma	Blood serum
Pietrzak et al. 2021 [8]	336	Cd (<0.57 * vs. >1.97 μg/L)	HR = 7.36; 95% CI: 2.14–25.25; *p* < 0.01	Lung	Blood
		Hg (<0.44 vs. >1.30 μg/L *)	HR = 1.55; 95% CI = 1.03–2.34; *p =* 0.04		

HR—hazard ratio; CI—confidence interval; *—reference group.

**Table 5:** Mechanisms of micronutrients in survival.

Mechanism of Action	Se	Zn
Generating oxygen free radicals/involved in oxidative stress/antioxidant	– Selenoproteins, thioredoxin reductase, and glutathione peroxidase reduce the number of free radicals [32,33]	– Zn/Cu superoxide dismutase [34,35,36]
Neoplastic growth	– Different proteins containing Se, such as thioredoxin reductase and glutathione peroxidase 1, 2, 3, 4, 6 [37,38,39]	– Induction of signaling pathway WNT/β-catenin suppresses the proliferation of cancer cells [40] – Inhibition of cell migration and invasion [41]
DNA repair	– Increase in tumor-suppressor protein p53 [32,42,43]	– Transcription and replication are regulated by zinc-finger proteins, which modulate the activity of DNA binding, including p53, AP-1, and NFκB [44,45,46,47,48]
Apoptosis and cell signaling	– Induction of apoptosis by p53 serines 20 and 37 [49,50,51,52,53,54]	– Apoptosis induction by pathway NFκB, AP-1, and ERK; dependent on H-Ras activation [41,44,47,55,56,57] – Activation of the signaling pathway WNT/β-catenin induces apoptosis in cells and inhibits cancer growth in osteosarcoma [40]
Maintaining DNA integrity in humans	– Selenomethionine reduces DNA damage [32]	– Prevention of DNA strand breaks [34,35,58,59]
Inflammation suppression	– Selenoenzymes have the ability to lower hydroperoxide compounds within the lipoxygenase and COX pathways, thus inhibiting the synthesis of PGL (proinflammatory ones) and LTR [32,33,60,61,62]	– Anti-inflammatory function of zinc-finger protein 36 (ZFP36) by downregulating pro-inflammatory cytokines, i.e., TNF-α [63] – Increase in production of IL-1β and IL-6, recognition of MCH-1, and suppression of NK cell cytotoxicity in the case of Zn deficiency [35]
Immune response enhancement	– Supplementing with Se (Na2SeO3) amplifies the immunological response by elevating the counts of cytotoxic lymphocytes and NK cells [64]	– Regulatory T cell function suppression by ZFP36L2 [63] – Granulocyte recruitment impairment, phagocytosis, chemotaxis, ROS generation, and epithelial cell–monocyte adhesion are regulated by Zn levels [35] – Deficiency results in decreased T cell immunity [65]
Protein kinase C inactivation	– The specific deactivation of PKC occurs through the interaction of its catalytic domain with selenometabolites, like CH3SeO2H, which is produced from membrane-bound CH3SeH and fatty acid hydroperoxides. This interaction hinders tumor promotion and the proliferation of cells [66]	
DNA methylation alteration	– DNA demethylation [67,68]	– Cofactor required for DNA methylation [69,70,71,72]
Angiogenesis inhibition	– Se promotes vascular endothelial cell apoptosis and inhibits angiogenesis through the MAPK pathway [73]	
Cell cycle blockage	– Cell cycle blockage caused by CH3SeH precursors [74]	– Cell cycle blockage properties of Zn (II)-phthalocyanine in photodynamic therapy [75] – Inhibition of cell cycle [41]
Telomere length—preserving telomere length leads to a decrease in the occurrence of age-related chronic diseases and cancers	– Antioxidant properties of Se reduce telomere attrition [76]	– Antioxidant properties of Zn preserve telomere length [77]
Regulation of thyroid function	– Se deficiency is associated with hypothyreosis, which is associated with increased survival, especially in older people [78] – Protein that includes a selenocysteine plays a role in the metabolic processes of thyroid hormones [79]	– Zn participates in the biosynthesis of thyroid hormones [80]
Cardiovascular disease	– Reduces levels of oxidized LDL, damage to DNA caused by oxidation, and the generation of deoxyguanosine [81] – Heart failure decreases survival [82]	– Low Zn levels lead to calcification of blood vessels [83] – Ischemia-reperfusion injury [84]

## Data Availability

Data supporting the reported results are available from the corresponding author upon request from all interested researchers.
